# New method for quantification of gasotransmitter hydrogen sulfide in biological matrices by LC-MS/MS

**DOI:** 10.1038/srep46278

**Published:** 2017-04-13

**Authors:** Bo Tan, Sheng Jin, Jiping Sun, Zhongkai Gu, Xiaotian Sun, Yichun Zhu, Keke Huo, Zonglian Cao, Ping Yang, Xiaoming Xin, Xinhua Liu, Lilong Pan, Furong Qiu, Jian Jiang, Yiqun Jia, Fuyuan Ye, Ying Xie, Yi Zhun Zhu

**Affiliations:** 1 Department of Clinical Pharmacology, Shuguang Hospital Affiliated to Shanghai University of Traditional Chinese Medicine, Shanghai, China; 2 State Key Laboratory of Quality Research in Chinese Medicine and School of Pharmacy, Macau University of Science and Technology, Macau; 3 Shanghai Key Laboratory of Bioactive Small Molecules, Department of Pharmacology, School of Pharmacy, Fudan University, Shanghai, China; 4 Department of Physiology, Hebei Medical University, Hebei, China; 5 Department of Physiology and Pathophysiology, Shanghai Medical College, Fudan University, Shanghai, China; 6 Institutes of Biological Sciences, Fudan University, Shanghai, China; 7 State Key Laboratory of Genetic Engineering, School of Life Sciences, Fudan University, Shanghai, China; 8 Department of Cardiothoracic Surgery, Huashan Hospital of Fudan University, Shanghai, China; 9 Analysis Center, School of Pharmacy, Fudan University, Shanghai, China; 10 Instrumental Analysis Center, Shanghai University of Traditional Chinese Medicine, Shanghai, China

## Abstract

Hydrogen sulfide exists widely in mammalian tissues and plays a vital role in physiological and pathophysiological processes. However, striking differences with orders of magnitude were observed for the detected hydrogen sulfide concentrations in biological matrices among different measurements in literature, which lead to the uncertainty for examination the biological relevance of hydrogen sulfide. Here, we developed and validated a liquid chromatography- mass spectrometry (LC-MS/MS) method for the determination of hydrogen sulfide in various biological matrices by determination of a derivative of hydrogen sulfide and monobromobimane named sulfide dibimane (SDB). ^36^S-labeled SDB was synthesized and validated for using as an internal standard. This method has been successfully used to measure hydrogen sulfide levels in a broad range of biological matrices, such as blood, plasma, tissues, cells, and enzymes, across different species. Moreover, a novel mode that hydrogen sulfide could loosely and non-covalently bind to human serum protein (HSA) and hemoglobin (HB) was revealed by using the developed method.

Hydrogen sulfide (H_2_S), which has a characteristic odor of rotten eggs, has historically been considered as a toxic gas and an environmental pollutant^1^. Since the discovery of endogenous hydrogen sulfide in the mammalian brain^2^, numerous studies have found that this compound plays a vital role in physiological and pathological processes, including neuromodulation^3^,^4^, blood vessel relaxation^5^, cardioprotection^6^, insulin release^7^, as well as the regulation of inflammation^8^, angiogenesis^9^,^10^, and energy production^11^. In addition to nitric oxide (NO) and carbon monoxide (CO), hydrogen sulfide is recognized as a unique but ubiquitous gasotransmitter^12^. Currently, there are ongoing studies as well as clinical trials investigating the therapeutic potential of hydrogen sulfide^13^,^14^.

Hydrogen sulfide exists as three forms in aqueous solution, i.e., hydrogen sulfide gas (H_2_S), hydrosulfide anion (HS^−^), and sulfide anion (S^2−^). However, in addition to these free forms (H_2_S, HS^−^, S^2−^), hydrogen sulfide also exists in other bound forms in the biological matrix, such as the acid labile, alkaline labile, and reducible forms, which are involved in releasing free hydrogen sulfide in response to a physiological stimulus^15^. Therefore, the promiscuous chemical properties make it difficult for accurate and reliable measurement of hydrogen sulfide in biological matrix^15^. Various methods have been established for endogenous hydrogen sulfide measurement, including using colorimetry^16^, gas chromatography^17^, electrodes selective for sulfide (ion-selective electrodes, ISEs)^18^, polarographic sensors^19^, fluorescent probes^20^, and high performance liquid chromatography (HPLC coupled with ultraviolet, fluorescence or electrochemical detection)^21^,^22^. However, these methods have obvious limitation, such as complex preparation processes, low specificity and sensitivity, and time-consuming procedures^15^. Moreover, striking differences with orders of magnitude were observed for the detected hydrogen sulfide concentrations using these methods, which lead to the uncertainty for examination the biological relevance of hydrogen sulfide^23^.

Due to the low molecular weight and simple chemical structure, hydrogen sulfide is not suitable for quantification directly by triple quadruple mass spectrometry and thus chemical derivatization were used before detection by LC-MS. Monobromobimane (MBB) is a common fluorescent reagent that reacts rapidly and completely with thiol groups^24^,^25^. Hydrogen sulfide has two nucleophilic substitution which could reacted with MBB to form a fluorescent derivative, sulfide dibimane (SDB) (Fig. 1a and Supplementary Fig. S1). Accordingly, a fluorescent detection coupled with high performance liquid chromatography (HPLC) has been developed and widely used to determine the hydrogen sulfide concentrations in biological samples in recent studies^21^,^26^,^27^,^28^. However, the specificity and sensitivity of this method is not sufficient to evaluate the endogenous thiol-containing compounds and other endogenous fluorescent compounds in biological samples^29^. Very few studies have been carried out with mass spectrometry technology to determination of hydrogen sulfide concentrations which exhibits excellent selectivity and sensitivity^30^. Moreover, a reliable quantitative measurement for hydrogen sulfide has not been established due to lack of a suitable internal standard^21^,^31^,^32^.

In this paper, we present a validated liquid chromatography-tandem mass spectrometry (LC-MS/MS) method with high sensitivity and specificity that can robustly measure hydrogen sulfide concentrations in various biological samples. Successfully using this method in human blood samples revealed a novel loosely bound form of hydrogen sulfide.

## Results

### Establishing a quantitative assay for hydrogen sulfide

**Figure 1:**
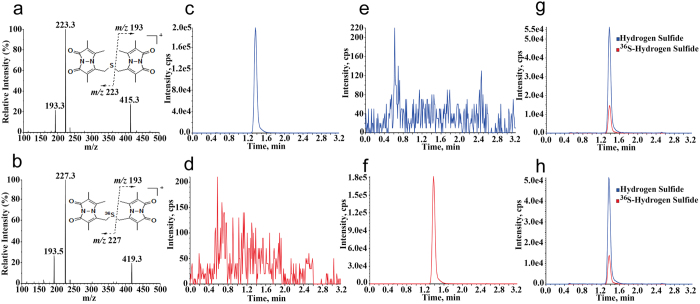
Analysis of hydrogen sulfide in different biological matrices. (**a**,**b**) The MS/MS spectrum of hydrogen sulfide derivatives, sulfide dibimane (SDB) (**a**) and ^36^S-labeled sulfide dibimane (^36^S-SDB) (**b**). (**c**,**d**) The selected reaction monitoring (SRM) chromatograms (*m/z* 415.3 to *m/z* 223.3) (**c**) or (*m/z* 419.3 to *m/z* 227.3) (**d**) for the sodium sulfide solution (5 μM). (**e**,**f**) The SRM chromatograms (*m/z* 415.3 to *m/z* 223.3) (**e**) or (*m/z* 419.3 to *m/z* 227.3) (**f**) for the ^36^S labeled-sodium sulfide solution (5 μM). (**g**) The combined SRM chromatograms of SDB (1.25 μM) and ^36^S-labeled SDB (0.313 μM) in an authentic standard solution. (**h**) The combined SRM chromatograms of SDB and ^36^S-labeled SDB (IS) in mice blank plasma. Data were collected using UPLC (Waters) coupled with a triple quadrupole mass spectrometer (AB Sciex 5500).

In this study, we aimed to develop a reliable LC-MS/MS method for measuring hydrogen sulfide in various biological matrices. Firstly, with the synthesized sulfide-MBB derivative SDB, we optimized the mass spectrometer conditions. In the positive ionization scan mode, a protonated molecule ([M + H]^+^, *m/z* 415) was present as precursor ion (Fig. 1a). Two major product ions at *m/z* 193 and *m/z* 223 were observed in the MS/MS spectrum, showing different C-S bond breakage in the SDB skeleton (Fig. 1a). The product ion *m/z* 193 is not composed of sulfur atom as reported in the literature^21^,^31^. It is a common fragment ion for all MBB-thiol derivatives. Using the ion of *m/z* 193 for analysis would potentially bring cross-talk interference caused by co-effluents in biological matrices. Fortunately, the product ion *m/z* 223 contains a sulfur atom. Furthermore, it exhibits a higher signal response than that of the ion *m/z* 193 (Fig. 1a). Thus, product ion *m/z* 223 were used for analysis of SDB.

Secondly, selecting a suitable internal standard (IS) is critical important for LC-MS/MS determination. The stable isotope labeled internal standard (SIL-IS) possessed identical physicochemical properties as analyte is always the best choice. As precursor ion and the selected product ion *m/z* 223 for SDB are composed of sulfur element, stable sulfur isotope labeled internal standard is thought to be an ideal IS. The natural abundances of four known stable sulfur isotopes are as following: ^32^S (95.02%), ^33^S (0.75%), ^34^S (4.21%), and ^36^S (0.02%). In previous literatures ^34^S-labeled SDB was widely used as a SIL-IS^32^. The major problem is that the mass difference between analyte and ^34^S-labeled SIL-IS is less than 3 mass units which makes the signal of isotope peaks of analyte interfering with that of IS^33^. Therefore, in this study, ^36^S-labeled SDB with maximum mass difference and minimum natural isotopic abundance was designed and synthesized as a SIL-IS. Compared with non-labeled SDB, the synthesized ^36^S-SDB showed differences of *m/z* (Δ(*m/z*) = 4) between their precursor ions and product ions (Fig. 1b). No interference between analyte (SDB) and IS (^36^S-labeled SDB) was observed during sample detection (Fig. 1c–f).

**Figure 2:**
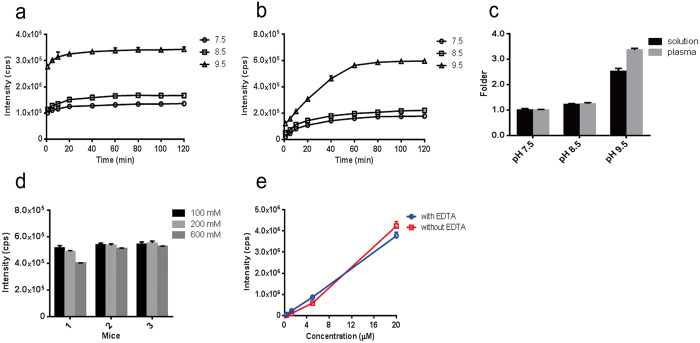
Optimization of MBB derivatization conditions. (**a**,**b**) Test for three pH values and incubation times in a sodium sulfide solution (1 μM) (**a**) and mice plasma (**b**). (**c**) Normalized responses at three pH values by the responses at pH 7.5 for either a sodium sulfide solution (1 μM) or mice plasma after 120 min of derivatization. (**d**) Comparison of the response for Tris-HCl (pH 8.5) buffer at three ionic strengths (100, 200, 600 mM) in mice plasma. (**e**) Comparison of linearity of response of four sodium sulfide levels (0.313, 1.25, 5 and 20 μM) with or without 2.0 mg/mL EDTA. Data are represented as the mean ± SD (n = 3).

Thirdly, to optimize the derivatization efficiency, we compared three conditions including pH value, reaction time, and buffer ionic strength. Sodium sulfide solutions (1 μM) or normal mouse plasma were adjusted to three pH values (pH 7.5, 8.5, and 9.5) by mixing with Tris-HCl buffer. More intense responses was observed in the time – response curves for both matrices with higher pH value, indicating that the alkaline condition would benefit the derivatization efficiency (Fig. 2a,b). To evaluate the hydrogen sulfide level under physiological conditions, a relatively mild pH value of 8.5 was chosen in this study. Another benefit of using pH 8.5 was that the weak alkaline condition could inhibit the H_2_S volatilization during sample processing. Moreover, after 120 min of derivatization, we noticed that there was an obvious increase in hydrogen sulfide in mouse plasma compared with that in the sodium sulfide solution (3.4-fold *vs* 2.5-fold higher, *P* < 0.01) at the highest pH value (pH 9.5), which implied that hydrogen sulfide was released from its potential forms (Fig. 2c). Although the MBB derivatization rate for hydrogen sulfide was faster in solution than that in plasma, after 60 min of derivatization, all of the products of hydrogen sulfide exceeded 90% of their maximum yield in solution and in mice plasma (Fig. 2a,b). Thus, the derivatization time in the sample process was set to 60 min. In addition, as the ionic strength of Tris-HCl buffer affects the derivatization efficiency and mass spectrometry responses, three buffers with different ionic strengths, i.e., 100, 200 and 600 mM, were compared. The ionic strength of 100 mM was chosen due to its higher response than that of other ionic strengths (Fig. 2d). Moreover, a common metal chelating agent, ethylene diamine tetraacetic acid (EDTA), was used in the sample preparation as hydrogen sulfide could react with trace amounts of metal ions in this circumstance. A good linear curve was also constructed by adding 2.0 mg/mL EDTA in the standard samples (*r* = 0.9976 *vs r* = 0.9436, Fig. 2e).

**Figure 3:**
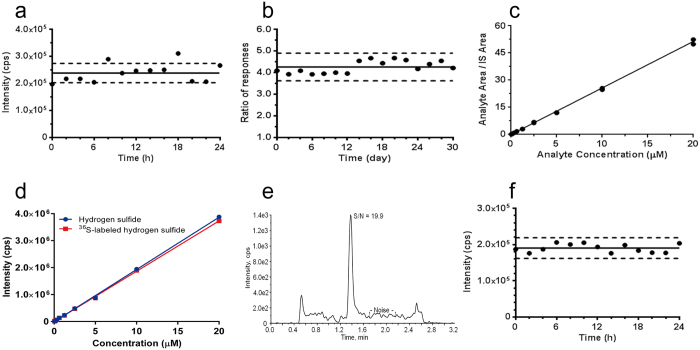
Validation of the robustness, signal-to-noise ratio and linearity of the assay. (**a**) Variance of responses for hydrogen sulfide in processed mice plasma without the internal standard (IS) over 24 h. (**b**) Variance of responses for hydrogen sulfide in processed mice plasma with the IS over 30 days. (**c**) The typical standard curve for hydrogen sulfide with the IS (*y* = 2.57*x* + 0.025, *r* = 0.9981). (**d**) Comparison of external standard curve between hydrogen sulfide and ^36^S-labeled hydrogen sulfide. (**e**) The signal-to-noise value for hydrogen sulfide at the lower limit of quantification (0.039 μM). (**f**) Stability of hydrogen sulfide in processed mice plasma under room light for 24 h (data were reconstructed by the IS response). Each point represents the average value of the triplicates. The dotted line represents ± 15% variation.

Then, method validation was performed following the guidelines for Bioanalytical Method Validation published by the Food and Drug Administration (FDA)^34^. The typical chromatograms of hydrogen sulfide and IS in mice plasma and other tissues are shown in Supplementary Fig. S3. No significant interferences were observed at the retention times of the analytes and ^36^S-labeled hydrogen sulfide with different biological matrices. The suitability of IS was further evaluated by determining the hydrogen sulfide content repeatedly in a mouse plasma sample within one month which was processed in the same manner (Fig. 3a,b). Without the IS correction, the variation for the MS response of hydrogen sulfide in plasma exceeded ± 15% of the mean response within 24 hours (Fig. 3a). But the response ratio of analyte to IS was stable for over 30 days by using ^36^S-SDB as the IS (Fig. 3b). All data were variable within ±10% of the mean value supporting the appropriateness of the IS for ensuring analytical accuracy. Calibration curves were prepared over the concentration range of 0.039 to 20 μM and were linear with regression coefficients >0.99 (Fig. 3c). The mean equation of the calibration curves generated during the validation was *y* = 2.57*x* + 0.025 (*r* = 0.9981). Meanwhile, a linear curve was constructed for the ^36^S-labeled hydrogen sulfide over the same concentration range for the following evaluation (Fig. 3d). The corresponding limit of detection (LOD) was 0.039 μM (Fig. 3e). The intra- and inter-day precision values for QC samples were less than 9.0%, and the accuracy was within ±9.1% (Supplementary Table S1). Although a minor absolute matrix effects were detected in different tissues (Supplementary Table S2), they would be compensated by the SIL-IS. Stability testing data indicated that the hydrogen sulfide derivative SDB in prepared samples were stable either putting short-term at room temperature or long-term at −20 °C (Supplementary Table S3). Although MBB is a light-sensitive agent, there are no significant decrease in the level of SDB after exposed to room light for 24 h (Fig. 3f), indicating that processed samples were no need to strictly avoid lights.

### Quantifying the production of hydrogen sulfide in neonatal rat cardiac ventricular myocytes and the budding yeast CSE enzyme

**Figure 4:**
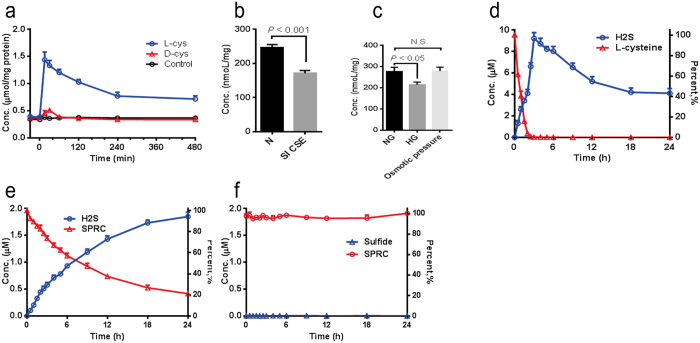
Determination of hydrogen sulfide in rat cardiac ventricular myocytes (NRCMs) and yeast CSE enzyme (CYS3). (**a**) Hydrogen sulfide production with two substrates, L-cysteine for CSE and D-cysteine for 3MST in NRCMs. (**b**) Hydrogen sulfide production with or without CSE siRNA in NRCMs. (**c**) Hydrogen sulfide levels with or without high glucose treatment in NRCMs. (**d**) Hydrogen sulfide production with L-cystathionine (CTT) in yeast CYS3. (**e**,**f**) Hydrogen sulfide production with S-propargyl-cysteine (SPRC) in yeast CYS3 without (**e**) or with (**f**) the propargylglycine (PAG) inhibitor.

Mammalian cardiomyocyte contains abundant cystathionine-γ-lyase (CSE), one of the hydrogen sulfide synthesize enzymes. With the developed method, we measured the production of hydrogen sulfide in primary neonatal rat cardiac ventricular myocytes (NRCMs) with L- or D-cysteine, respectively. L-cysteine is an endogenous substrate for CSE, whereas D-cysteine was recently reported as a substrate for 3-mercaptopyruvate sulfurtransferase (3MST), another synthesize enzyme of hydrogen sulfide^35^. Here, we found that the hydrogen sulfide level in NRCMs immediately increased upon addition of L-cysteine or with D-cysteine (Fig. 4a). However, only L-cysteine had significant and continuous influences on the production of hydrogen sulfide (Fig. 4a). Moreover, the CSE expression was significantly reduced in NRCMs with transfection of CSE siRNA (Supplementary Fig. S4) in consistent with 31% decrease in the concentration of hydrogen sulfide (*P* < 0.001) (Fig. 4b). In addition, as glucose stimulation has been reported to be involved in the H_2_S-producing activity of CSE in INS-1E cells^36^, we observed that the NRCMs treated with a high glucose level for three days have significantly decreased hydrogen sulfide production (by 22%) compared with the control group (*P* < 0.05) (Fig. 4c).

Yeast CSE enzyme (CYS3) has similar conserved domains and activities as human CSE^37^. We incubated purified yeast CYS3 with L-cystathionine and observed that the hydrogen sulfide production increased rapidly whereas the amount of L-cystathionine (CTT) decreased accordingly (Fig. 4d). To further explore the using of our developed method, the enzymatic kinetics of S-propargyl-cysteine (SPRC), a novel synthetic sulfur-containing candidate were investigated with our method. SPRC could elevate the hydrogen sulfide level in animals and in isolated tissues, and exhibits several pharmacological activities that are similar to those of hydrogen sulfide^10^,^38^. Moreover, SPRC is structurally analogous to L-cysteine, and has been assumed as a CSE substrate, although there are no convincing enzymatic data^10^. In this study, we observed that SPRC elimination was accomplished with increased level of hydrogen sulfide in the CYS3 mixture. The average catalytic capability of CYS3 on SPRC was 0.89 pmol/mg/min in the incubation mixture, which was approximately 13 times lower than that of CTT (11.5 pmol/mg/min) (Fig. 4d,e). Furthermore, the specific CSE inhibitor propargylglycine (PAG) completely inhibited the hydrogen sulfide production of CYS3 by using SPRC as a substrate (Fig. 4f). Thus, we could proposed that human CSE possesses a similar function as that of CYS3 in SPRC metabolism with hydrogen sulfide as one of its products. In sum, these findings suggested that our method could be used to evaluate the changes in the hydrogen sulfide level in both cultured cells and enzymes.

### Quantifying hydrogen sulfide in mice or rat plasma and tissues

**Figure 5:**
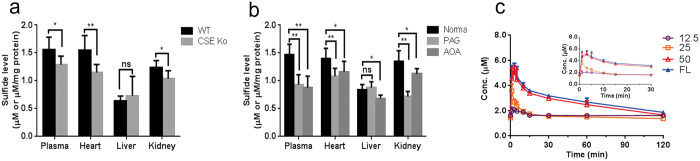
Determination of hydrogen sulfide in mice tissues. (**a**) Hydrogen sulfide concentration in different tissues in both wild-type mice (*CSE*^*+/+*^) and CSE knockout mice (*CSE*^−/−^). (**b**) Hydrogen sulfide concentration in different tissues in wild-type mice with propargylglycine (PAG, CSE inhibitor) or amino-oxyacetate (AOA, CBS inhibitor) treatment. (**c**) Hydrogen sulfide concentrations in rat plasma after a single intraperitoneal injection of 12.5, 25 and 50 μM NaHS solution using the LC-MS-MS or HPLC-FL method. * represents *P* < 0.05; ** represents *P* < 0.01; ns represents not significant.

To evaluate the endogenous hydrogen sulfide baseline in the most relevant tissues including plasma, heart, liver, and kidney with the developed method. CSE knockout mice (*CSE*^−/−^) the most important models for hydrogen sulfide biological studies or wild-type mice (*CSE*^*+/+*^) were used for comparing. We observed that the hydrogen sulfide levels in all determined tissues from knockout mice, except for liver, decreased significantly compared with wild-type mice (Fig. 5a). Another study was conducted with administration of two inhibitors for endogenous hydrogen sulfide synthetases, namely PAG as a CSE inhibitor, and amino-oxyacetate (AOA) as the cystathionine-β-synthase (CBS) inhibitor, respectively in wild-type mice. Significant decrease of hydrogen sulfide levels in all determined tissues for both treatments were observed except for the level in liver after giving PAG for three days (Fig. 5b).

### Comparison of the developed LC-MS/MS method with HPLC-FL method

To further verifying our results determined with the developed LC-MS/MS method, after intraperitoneal injection of NaHS we compared the plasma hydrogen sulfide levels in rat determined by our developed method and reported HPLC-FL method which is a newly confirmed method that has been used to determine hydrogen sulfide in certain biological samples, e.g., blood and plasma^31^. Both the HPLC-FL method and our method obtained similar data (Fig. 5c). Moreover, the concentration-time curves for the two methods were very similar, although the values obtained by our method were slightly lower than those by HPLC-FL. This difference might be due to the interferences caused by other biological thiol derivatives using HPLC-FL for determination (Supplementary Fig. S2).

### Quantifying hydrogen sulfide in human blood

**Figure 6:**
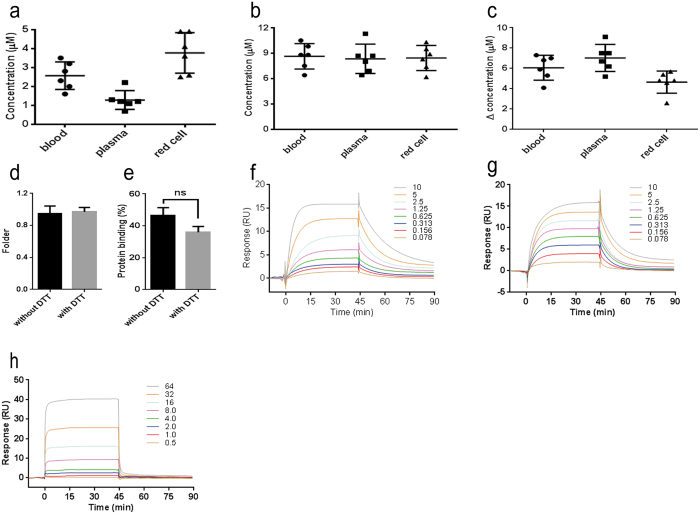
Determination of hydrogen sulfide in human blood (n = 6). (**a**) The baseline hydrogen sulfide in human blood, plasma, and red cells. (**b**) The hydrogen sulfide levels in human blood, plasma, and red cells pretreated with DTT. (**c**) The DTT reducible hydrogen sulfide levels in human blood, plasma, and red cells. (**d**) The ratio between calculated hydrogen sulfide and determined hydrogen sulfide levels in human blood with or without DTT. (**e**) The protein binding rate of hydrogen sulfide in human plasma pretreated with or without DTT. (**f**) The typical blank subtracted sensorgrams of hydrogen sulfide to human serum protein (HSA) with the hydrogen sulfide levels ranging from 0 to 10 mM. (**g**) The typical blank subtracted sensorgrams of hydrogen sulfide to human hemoglobin (HB) with the hydrogen sulfide levels ranging from 0 to 10 μM. Data are represented as the mean ± SD, except for the sensorgrams. (**h**) The blank subtracted sensorgrams of (*S*)-warfarin to human serum protein (HSA) with hydrogen sulfide levels ranging from 0 to 64 nM.

The quantification of endogenous hydrogen sulfide in blood, plasma, and red cells using a single method remains an intriguing challenge. In this study, our developed method was used to measure hydrogen sulfide levels in human samples to provide reliable data for the hydrogen sulfide baseline. Among six healthy volunteers, the mean hydrogen sulfide baseline values for blood, plasma, and red cells were 2.6 ± 0.7 μM, 1.3 ± 0.5 μM, and 3.8 ± 1.0 μM, respectively (Fig. 6a), indicating that hydrogen sulfide was distributed more abundantly (3.1-fold higher, *P* < 0.01) in red cells than in plasma. In addition, dithiothreitol (DTT), which has a similar redox potential to that of endogenous redox molecules, e.g., glutathione, was used to increase hydrogen sulfide releasing from its bound forms by cleavages of disulfide bonds. Interestingly, the determined hydrogen sulfide (also called physiological available hydrogen sulfide) levels in human blood, plasma, and red cells were equally distributed, with values ranging from 8.3 to 8.6 μM (*P* = 0.94) (Fig. 6b). The DTT reducible hydrogen sulfide level could be calculated by subtracting the hydrogen sulfide baseline from the determined hydrogen sulfide pretreated with DTT. In contrast to the baseline hydrogen sulfide, the DTT reducible hydrogen sulfide was distributed more abundantly in plasma (1.5-fold higher, *P* < 0.01) than in red cells (Fig. 6c). Moreover, we calculated the hydrogen sulfide level in blood by combing the hydrogen sulfide levels in plasma and in red cells with a theoretical volume ratio (at blood: plasma: red cell = 1.0: 0.55: 0.45 with ignoring other very low-volume components in blood, such as leukocytes). The calculated hydrogen sulfide levels were consistent with the determined hydrogen sulfide levels in blood in the presence (0.97 ± 0.05-fold higher, *P* = 0.78) or absence of DTT (0.95 ± 0.10-fold higher, *P* = 0.73) (Fig. 6d).

Next, we used our method to investigate the protein binding rate of hydrogen sulfide in human plasma using a traditional ultrafiltration method^39^. By calculating the concentrations of hydrogen sulfide in both the upper and lower layer samples in an ultrafiltration tube, the protein (MW ≥ 10 kDa) binding rate was calculated as 46.6 ± 11.9% (Fig. 6e), which means there are 1.3 ± 0.4 μM hydrogen sulfide bound in human plasma protein. Treatment with DTT significantly increased the free hydrogen sulfide level in plasma (approximately 7.0-fold higher) (Fig. 6c), while the protein binding rate of hydrogen sulfide was just slightly reduced by 10.6% (Fig. 6e). These findings indicated that a large portion of the released hydrogen sulfide might bind to the plasma proteins. To verify this hypothesis, we investigated the direct interaction between hydrogen sulfide and immobilized human serum album (HSA) or hemoglobin (HB) by applying Biacore’s surface plasmon resonance (SPD) technology^40^. NaHS solutions ranging from 0 to 10 mM were injected over a HSA or HB surface on chips. In all samples, the SPD responses were concentration dependent with rapidly achieved equilibria, and dissociation of the complexes was complete within tens of seconds, indicating that the bindings were readily reversible (Fig. 6f,g). No regeneration of the surface was required between injections, and no carryover was observed in buffer blanks injected intermittently throughout the analysis. However, compared with (*S*)-warfarin, a typical high-affinity binding compound for HSA, hydrogen sulfide exhibited a slower binding profile during both the absorption and dissociation phases for both HSA and HB, which implied a moderate capability of protein binding (Fig. 6h).

## Discussion

Biologists around the world have studied hydrogen sulfide for decades, and many more questions have been asked than answered^15^. Emerging studies have demonstrated that hydrogen sulfide plays a vital role in biological activities^15^. Due to the importance of the biological levels of hydrogen sulfide, several measurement methods have been established. However, the actual hydrogen sulfide level in biological matrices remains unclear because little consensus was achieved at the concentrations of hydrogen sulfide^14^. For example, the hydrogen sulfide baseline in mammalian blood is controversial, as it ranges from undetectable to hundreds of micromoles, even though similar measurement methods were used^14^. An accurate and sensitive determination method for convenient application to different biological matrices is needed.

With a sulfur-containing product ion for sulfide dibimane (SDB), which is a derivative of hydrogen sulfide and monobromobine, we developed and validated a LC–MS/MS method for the determination of hydrogen sulfide in various biological matrices. A stable 36-sulfur isotope labeled internal standard was used to ensure sensitivity and feasibility. The high-throughput capability (approximately 300–400 samples per day) of this method makes it suitable for large-scale analysis. Moreover, if be necessary this method could easily expand the detectable limits to the sub-nanomolar scale either by raising up the sample consumption or injection volume for analysis.

We observed that a modest level (approximately several μM) of hydrogen sulfide exists in blood and other tissues for different species across mice to human, which is in accordance with the literature^41^. However, the question raised out from results is about why the normal biological matrices did not emit the characteristic odor of hydrogen sulfide. This finding is opposite to the fact that humans have an acute sense of smell for H_2_S with levels as low as 1 μM Na_2_S solution being perceptible^42^. Several studies attribute this finding to the existence of a potential hydrogen sulfide pool, such as iron-sulfur clusters and sulfanes, in which hydrogen sulfide is bound covalently^26^,^43^,^44^. As no direct evidence supports the stripping of hydrogen sulfide by MBB from the bound forms under mild conditions, we assumed that there was another loosely bound form of hydrogen sulfide in the biological matrices that could also mediate the H_2_S level. Using a traditional protein binding experiment employing ultrafiltration with or without the reductant DTT, we observed that hydrogen sulfide exhibited nearly half percent binding rate to human plasma proteins (MW ≥ 10 kDa). To verify this result, a direct affinity assay was conducted between hydrogen sulfide and two major blood/plasma proteins, serum albumin (HSA) and hemoglobin (HB), on chips using the Biacore SPD system. We found that hydrogen sulfide could rapidly and reversibly bind to HSA and HB with weaker affinity than that of warfarin, a typical high-affinity protein binding molecule. Although the underling mechanism for the binding is unclear, it is proposed that hydrogen sulfide in biological matrices exists at least partly in the loosely and reversibly bound form to various abundant proteins. Thus, the free hydrogen sulfide level in biological matrices may be lower than that detectable by the nose. Other authors have reported that dissolved H_2_S was present in trace amounts (approximately 15 nM) in blood and tissues, as measured by polarographic measurements^19^ and gas chromatography^17^, which is consistent with our findings. In addition, distinct from the constitutively bound forms that primarily exist as DTT-released forms (such as sulfanes, polysulfides, or persulfides)^45^,^46^, the binding mechanism may be non-covalent and reversible, which is similar to that of many small molecules (such as fatty acids, hormones, amino acids, cations, and drugs) to ensure their distribution throughout the body. This mechanism could explain either the decreasing dissolved hydrogen sulfide level, which reduces toxicity, or its prolonged existence, thus resulting in physiological/pathological effects. These factors should be studied in the future.

## Methods

### Materials

Sodium sulfide was purchased from Alfa Aesar, Ltd. (Ward Hill, MA, U.S.A). Monobromobimane, D,L-propargylglycine (PAG) and amino-oxyacetate (AOA) were purchased from Sigma Co., Ltd (St. Louis, MO, U.S.A). 36-Sulfur powder was purchased from Campro Scientific GmbH (Berlin, German). Acetonitrile was obtained from Merck (Darmstadt, Germany). CM5 sensor chips were purchased from GE Healthcare AB (Uppsala, Sweden). Other reagents and solvents were of the highest quality available.

### Synthesis of sulfide dibimane and ^36^S-labeled sulfide dibimane derivatives

Stable isotope-labeled sodium sulfide (Na_2_^36^S) was synthesized according to the literature^47^. Briefly, the 36-sulfur powder, sodium, naphthalene, and tetrahydrofuran (THF) were mixed and then refluxed for 12 h; at that time, the color of the mixture changed from yellow to white. The mixture was filtered and eluted, and the dissolved portion containing ^36^S-sodium sulfide was dried and stored until use. All reaction procedures were conducted under a nitrogen atmosphere. Then, sulfide dibimane (SDB) and ^36^S-labeled sulfide dibimane (IS) were synthesized using sodium sulfide and ^36^S-sodium sulfide, respectively, with monobromobimane, according to the literature^21^. Using the reaction proportion of 1: 2, the sodium sulfide (or ^36^S-sodium sulfide) solution was added dropwise with stirring at room temperature to a monobromobimane solution dissolved in acetonitrile containing HEPES buffer (100 mM, pH 8.0). The solution was extracted with methylene chloride. The organic layers were collected and evaporated under a nitrogen stream. The residue was redissolved and purified using reversed-phase HPLC.

### Preparation of stock solutions, calibration samples, and quality controls

Stock solutions were freshly prepared by dissolving the reference standards in Tris-HCl buffer (100 mM, pH = 8.5) at a concentration of 1.0 mM for both analyte and IS in the glove box (MIKROUNA, Shanghai, China) under hypoxic conditions. A series of working standard solutions was obtained by further diluting the stock solutions in Tris-HCl buffer (100 mM, pH = 8.5). The IS working solution (1 μM) was obtained by diluting the stock solution in Tris-HCl buffer (100 mM, pH = 8.5). Calibration standards were prepared by spiking the appropriate amounts of the standard solutions into 500 μl of Tris-HCl buffer (100 mM, pH = 8.5) to yield final concentrations of 0.039, 0.078, 0.156, 0.312, 0.625, 1.25, 2.5, 5, 10, and 20 μM. The QC samples were similarly prepared at concentrations of 0.078, 2.5 and 18 μM for the low, medium and high concentration QC samples, respectively. All solutions were freshly prepared before use.

### Derivatization

A 30 μL sample (e.g., plasma) was spiked with 10 μL Tris-HCl buffer (100 mM, pH = 8.5), 10 μL EDTA solution (2.0 mg/mL) and 70 μL MBB solution (20 mM). After the mixture was vortexed for 60 min, the derivatization reaction was stopped by adding 10 μL 20% formic acid. Then, a 10 μL ^36^S-labeled SDB solution (IS) was added. After the mixture was subjected to centrifugation at 12,000 × rpm for 10 min, 1 μL of the supernatant was injected into the LC–MS/MS system for analysis.

### LC-MS/MS analysis

Chromatography was performed using a Waters Acquity UPLC (Milford, MA, USA) equipped with a Kinetex XB-C18 column (50 mm × 2.1 mm, 2.6 μm) from Phenomenex (Torrance, CA, USA) maintained at 40 °C. The components were eluted by a gradient of A) 0.08% formic acid in water and B) acetonitrile. The gradient conditions of the mobile phase over 3.2 min were as follows: 0–1.4 min, 20% B; 1.4–2.4 min, 20–80% B; 2.4–2.8 min, 80% B; 2.8–2.9 min, 80–20% B; and 2.9–3.2 min, 20%B. The flow rate was 0.6 mL/min. The autosampler was maintained at 4 °C throughout the analyses.

The Waters Acquity UPLC was coupled with an AB Sciex API 5500 triple quadrupole mass spectrometer (Foster City, CA, Canada). Positive ionization mode for electrospray ionization source were used for detection. Nitrogen was used for the curtain and collision gas. Data were collected in selected reaction monitoring (SRM) mode using transitions of *m/z* 415.1 → *m/z* 223.3 (SDB) and *m/z* 419.1 → *m/z* 227.3 (^36^S-labeled SDB). The dwell time used was 150 ms per channel with a 5 ms pause between mass transitions. Optimized MS acquisition parameters were as follows: collision gas (CAD) 10 psi, curtain gas (CUR) 15 psi, ion source gas 1 (GS1) 50 psi, ion source gas 2 (GS2) 50 psi, ion spray voltage (IS) 5500 V, source temperature 500 °C, declustering potential (DP) 113 eV, entrance potential (EP) 10 eV, collision energy (CE) 32 eV, and collision cell exit potential (CXP) 10 eV. The data were acquired and processed using AB Sciex Analyst 1.5 Software (Foster City, CA, Canada).

### Validation of the analytical method

The method was validated for different biological matrices, including plasma, tissues (i.e., heart, liver, kidney), and cultured cells. Validation parameters such as selectivity, linearity, precision, accuracy, recovery, matrix effects, and stability were investigated^34^. Considering that hydrogen sulfide is present in all sources of biological matrices, blank biological samples could not be found for direct method validation. Alternatively, we used the surrogate analyte approach in which structurally identical compounds are used. In this study, ^36^S-labeled hydrogen sulfide (also used as an internal standard) and a quantitative linear curve for ^36^S-labeled hydrogen sulfide only were used. Selectivity was investigated by the comparison of blank samples from six individual mice to the spiked samples. Linearity was assured by adding increasing amounts of sulfide or ^36^S-labeled sulfide to pure water. Calibration standards were prepared and analyzed in duplicate on three consecutive days. The peak area ratio of analyte to IS versus analyte concentration was used to construct calibration curves. The calibration curves were fitted via a 1/x^2^ weighted linear least-squares regression model. The lower limit of quantification (LLOQ) was determined based on a signal-to-noise ratio of at least 10:1. Similarly, another linear calibration curve without IS was established by adding ^36^S-labeled sulfide for the purpose of a surrogate approach to validate the accuracy, precision, recovery, matrix effects, and stability.

To evaluate accuracy and precision, quality control (QC) samples were prepared for ^36^S-labeled sulfide in different matrices. The concentration of QC samples covered the low, medium, and high analytical range. Intra-day and inter-day accuracy and precision were evaluated by comparing the measured concentrations in the QC samples (six replicates of the QC samples per day) with the respective nominal concentrations, expressed in terms of relative error (RE) and relative standard deviation (RSD), respectively. Recovery was evaluated by comparing the peak areas obtained from the sample preparation from different matrices and samples without preparation. Matrix effects were evaluated by comparing the average responses of the surrogate analyte in the post-treatment solution with the corresponding average responses of solution-only samples. The stability of the hydrogen sulfide derivative was examined by analyzing replicates (n = 6) of the three levels of QC samples with respect to post-preparative, freeze–thaw stability, and long-term stability.

### Western blot analysis

Frozen left ventricle tissues were lysed with ice-cold RIPA buffer. Proteins were extracted and quantified using BCA reagent (Shen Neng Bo Cai Corp., Shanghai, China). Protein samples were separated on 10% SDS-PAGE gels and transferred to polyvinylidene fluoride (PVDF) membranes (Millipore, Bedford, MA, USA). The membranes were blocked with 5% non-fat milk at room temperature for 1 h and then incubated with antibodies directed against CSE, CBS, 3-MST, Collagen I, Bax, Bcl-2 (Santa Cruz Biotechnology, Santa Cruz, CA, USA), Collagen III (Abcam, Cambridge, MA, USA) and GAPDH at 4 °C overnight. After the membranes were washed with TBST, they were incubated with horseradish peroxidase-conjugated secondary antibodies at room temperature for 1 h. Specific bands were detected with SuperSignal^®^ West Pico Chemiluminescent Substrate (Thermo Scientific-Pierce, Rockford, IL, USA).

### HPLC fluorescence detection

The fluorescence measurement was conducted according to the literature with slight modifications^29^. Samples were processed with monobromobimane (MBB) as previously described. The hydrogen sulfide derivative, sulfide dibimane (SDB), was measured using an Agilent 1100 HPLC (Agilent Technologies, Santa Clara, CA, USA) equipped with a fluorescence detector (ex: 390 nm and em: 475 nm) and an Agilent eclipse XDB-C18 column (4.6 × 250 mm, 5 μm). The elution solvents were water containing 0.1% TFA (A) and acetonitrile containing 0.1% TFA (B). The mobile phase composition (A/B; v/v) was 90:10 at 0 min, 65:35 at 6 min, 45:55 at 17 min, 30:70 at 24 min, 10:90 at 25 min, 10:90 at 27 min and 90:10 at 30 min (isocratic until 35 min). The flow rate was 0.6 mL/min at room temperature, and the injection volume was 5 μL.

### Ethics

The use of animals was approved by the Animal Care and Experimentation Committee of Fudan University (Shanghai, China). All animals received human care in accordance with the Guide for the Care and Use of Laboratory Animals published by the National Institutes of Health (NIH publication no. 85–23, revised 1996). The use of human specimens for scientific purposes was approved by the Ethics Committee of Shuguang Hospital Affiliated to Shanghai University of Traditional Chinese Medicine (Shanghai, China). Written informed consent was obtained from each people prior to conducting research. The study was performed in accordance with all applicable China’s laws, regulations and guidelines (including GCP guideline) relating to the protection of human subjects as volunteers, as well as all proposed and published regulations of the Declaration of Helsinki.

### Hydrogen sulfide production in rat myocytes

Primary neonatal rat cardiac ventricular myocytes (NRCMs) were collected as previously described with some modifications^48^. The ventricles of newborn Sprague-Dawley rats (1–3 days old) regardless of sex were minced and digested with 0.125% trypsin. Isolated NRCMs were cultured in Dulbecco’s modified Eagle’s medium/F-12 (DMEM/F12, Life Technologies, Carlsbad, CA, USA) with the following supplements: L-glutamine (4 mM), streptomycin (100 μg/mL), penicillin (100 IU/mL), 5-bromo-2’-deoxyuridine (0.1 mM), and fetal bovine serum (10% v/v). The incubation conditions were maintained at 37 °C with 5% CO_2_. To observe the changes of hydrogen sulfide production, L- or D-cysteine (5.5 mmol/L), normal glucose (5.5 mmol/L) or high glucose (33 mmol/L) and CSE siRNAs (Invitrogen, Carlsbad, CA, USA) were added.

### Hydrogen sulfide production in purified budding yeast CSE protein (CYS3)

The yeast CYS3 protein was produced in our laboratory according to the literature^37^. Briefly, the CYS3 gene was first amplified using PCR from *Saccharomyces cerevisiae* S288C gDNA. Then, the purified PCR product was subcloned into E. coli expression vector pTrc99A (Pharmacia Biotech, Uppsala, Sweden). The constructed plasmid (pTrc-CYS3) was transformed into E. coli cells, and the cells were cultured with kanamycin. Once the sequence of the plasmids was confirmed, the cells were cultured in large conical flasks (5 L) to obtain a large number of cells. The lysate of cells was transferred onto a nickel column, and the SUMO enzyme was used to cut the SUMO-tag. The CYS3 protein was purified using an ÄKTA protein purifier (GE Healthcare, Uppsala, Sweden). The protein was tested for the presence of A280, and the number was 2.3 (3.76 g/L). Incubations were performed in 950 μL of 473 μM SPRC (or CTT, a specific substrate for both CSE and CYS3) and 50 μL of the purified protein solution (90 μM) for a series of indicated times (0 to 24 h). Inhibition studies were conducted as described above with the additional CSE inhibitor PAG (473 μM). The consumption of SPRC and CTT was measured according to the literature^49^,^50^.

### Preparation of plasma and other tissues in CSE wild-type and knockout mice

CSE wild-type (CSE^+/+^) and knockout mice (CSE^−/−^) with C57BL/6 genetic background (4–5 weeks old, 15–20 g weight) were obtained from the Shanghai Research Center for Model Organisms (Shanghai, China). The genotype was determined by two PCR reactions according to the literature^51^. The inhibition effect on endogenous hydrogen sulfide in both wild-type and knockout mice was evaluated using D,L-propargylglycine (PAG) or amino-oxyacetate (AOA) according to the literature^35^. Mice were injected with equal volumes of phosphate buffered saline (PBS), PAG (33.9 mg/kg), or AOA (17 mg/kg) for three days. Mice were then sacrificed, and plasma and other tissues were collected for measurement as the literature^28^. Hydrogen sulfide concentrations in tissues were adjusted by dividing by the protein concentrations, which were expressed as μmol/g of protein.

### Preparation of human blood

Six healthy male volunteers (age: 22 ± 2 year, weight: 72 ± 5 kg) without a history of smoking were enrolled in the study after providing informed consent. The volunteers fasted the entire night, and then, whole blood samples were collected via cubital veins and transferred to EDTA-containing tubes. One part of the whole blood sample was centrifuged at 3,500 × *g* for 5 min to separate the plasma and red cells. While all the samples (including blood, plasma, and red cells) were collected, a 30 μL sample from each volunteer and 10 μL 20 mM DTT were added to a 1.5-mL tube. The tube was kept in a 37 °C water bath for 15 min. Another part of the samples was prepared without DTT. The mixture was prepared as described above.

### Plasma protein binding of hydrogen sulfide

Ultrafiltration was used to separate the protein-bound hydrogen sulfide from the unbound hydrogen sulfide in human plasma using the Amicon Centrifree^®^ Filter System (10 kDa molecular weight cut off) (Amicon Inc., Beverly, MA, USA). Freshly collected human plasma (0.5 mL) was added to a filter tube and centrifuged at 3,000 × *g* for 20 min (note: the volume of the lower layer of sample in the tube did not exceed 50 μL, i.e., one-tenth of the added volume). The upper and lower samples were collected for LC-MS/MS analysis. The fraction of protein binding was calculated as


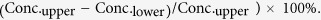


### Biacore analyses

Interaction analyses between hydrogen sulfide and two human plasma proteins, HSA and hemoglobin (HB), were performed using a Biacore T200 instrument (GE Healthcare, Uppsala, Sweden). Both HSA and HB were dissolved in 10 mM sodium acetate (pH 5.2). Using a flow rate of 20 μL/min, the surface of the flow cell with CM5 chips (GE Healthcare, Uppsala, Sweden) was activated for 7 min using a 1:1 mixture of 0.1 M *N*-hydroxysuccinimide (NHS) and 0.1 M *N*-ethyl-*N*-(3-dimethylaminopropyl)-carbodiimide (EDC), and then, 50 mg/mL HSA or HB was injected for 7 min. The residual activated groups on the surface were blocked by a 7-min injection of 1 M ethanolamine (pH 8.0), which was followed by three 10-s injections of 50 mM sodium hydroxide to remove any non-covalently bound HSA or hemoglobin. Finally, 11,000 ± 1,500 response units (RU) of HSA or HB were immobilized. Sodium hydrosulfide hydrate (Sigma, St. Louis, MO, USA) was used to freshly prepare a dissolved hydrogen sulfide solution before the experiment with a running buffer (5 × PBS). The dissolved hydrogen sulfide solution was injected in triplicate over the HSA or hemoglobin and reference surfaces ranging from 2 to 2,000 μM at a flow rate of 30 μL/min. The hydrogen sulfide/protein complex was allowed to associate and dissociate for 45 s each, and the surfaces were washed with running buffer for 10 s between each sample injection. (*S*)-warfarin was used as the positive control, and dissolved hydrogen sulfide solutions placed in a fume hood for 30 min were used as negative controls.

### Statistics

The results are expressed as the mean ± SD. Statistical analysis was performed using an SPSS software package, version 13.0 (SPSS, Inc., Chicago, IL, USA). The results for three or more groups were compared using one-way ANOVA followed by the Student-Newman-Keuls test. Comparisons between two groups were made using the two-tailed Student’s *t*-test. *P* < 0.05 was considered significant.

## Additional Information

**How to cite this article:** Tan, B. *et al*. New method for quantification of gasotransmitter hydrogen sulfide in biological matrices by LC-MS/MS. *Sci. Rep.* **7**, 46278; doi: 10.1038/srep46278 (2017).

**Publisher's note:** Springer Nature remains neutral with regard to jurisdictional claims in published maps and institutional affiliations.

## Supplementary Material

Supplementary Information
