# Supplementary material for: New method for quantification of gasotransmitter hydrogen sulfide in biological matrices by LC-MS/MS
Source: Sci Rep. 2017 Apr 13;7:46278. doi: 10.1038/srep46278 (PMC5390247; doi:10.1038/srep46278)
Supplement: Supplementary Information [file srep46278-s1.pdf]

New method for quantification of gasotransmitter hydrogen sulfide in biological matrices by LC-MS/MS

Bo Tan[1, 2, 3], Sheng Jin [4, 5], Jiping Sun[5], Zhongkai Gu[6, 7], Xiaotian Sun[8], Yichun Zhu[5], Keke Huo[7], Zonglian Cao[9], Ping Yang[9], Xiaoming Xin[3], Xinhua Liu[3], Lilong Pan[3], Furong Qiu[1], Jian Jiang[1], Yiqun Jia[10], Fuyuan Ye[10], Ying Xie[2, *], Yi Zhun Zhu[2, 3, *]

Supplementary Figure S1: Derivatization of hydrogen sulfide with monobromobimane.

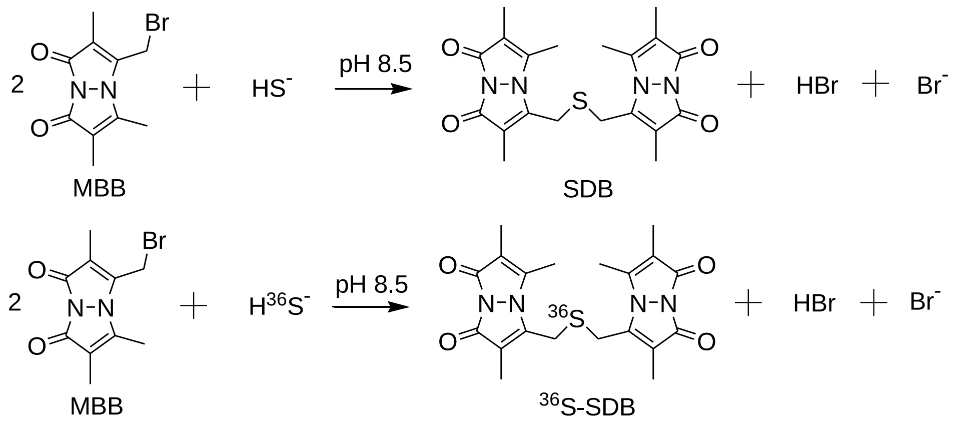

One molecule of hydrogen sulfide or ^{36}S-labeled hydrogen sulfide is derivatized with two molecules of monobromobimane to form sulfide dibimane (SDB) or ^{36}S-labeled sulfide dibimane (^{36}S-SDB).

Supplementary Figure S2: Analysis of hydrogen sulfide in rat plasma using liquid chromatography - fluorescence (HPLC-FL) detection without sample purification.

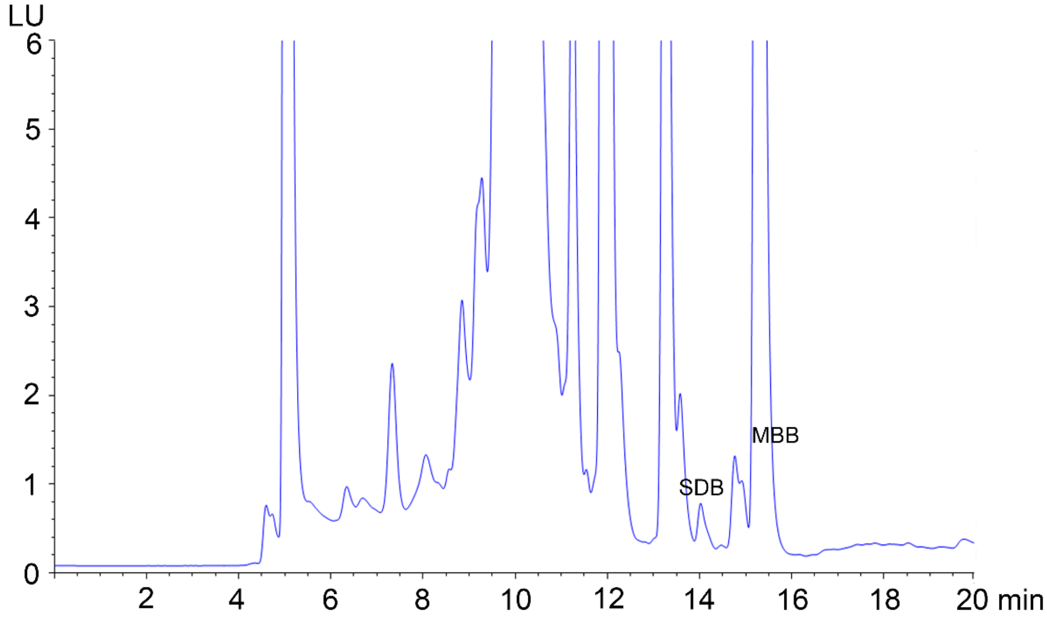

Supplementary Figure S3: Analysis of hydrogen sulfide and the internal standard (IS) in different mice matrices using LC/MS/MS.

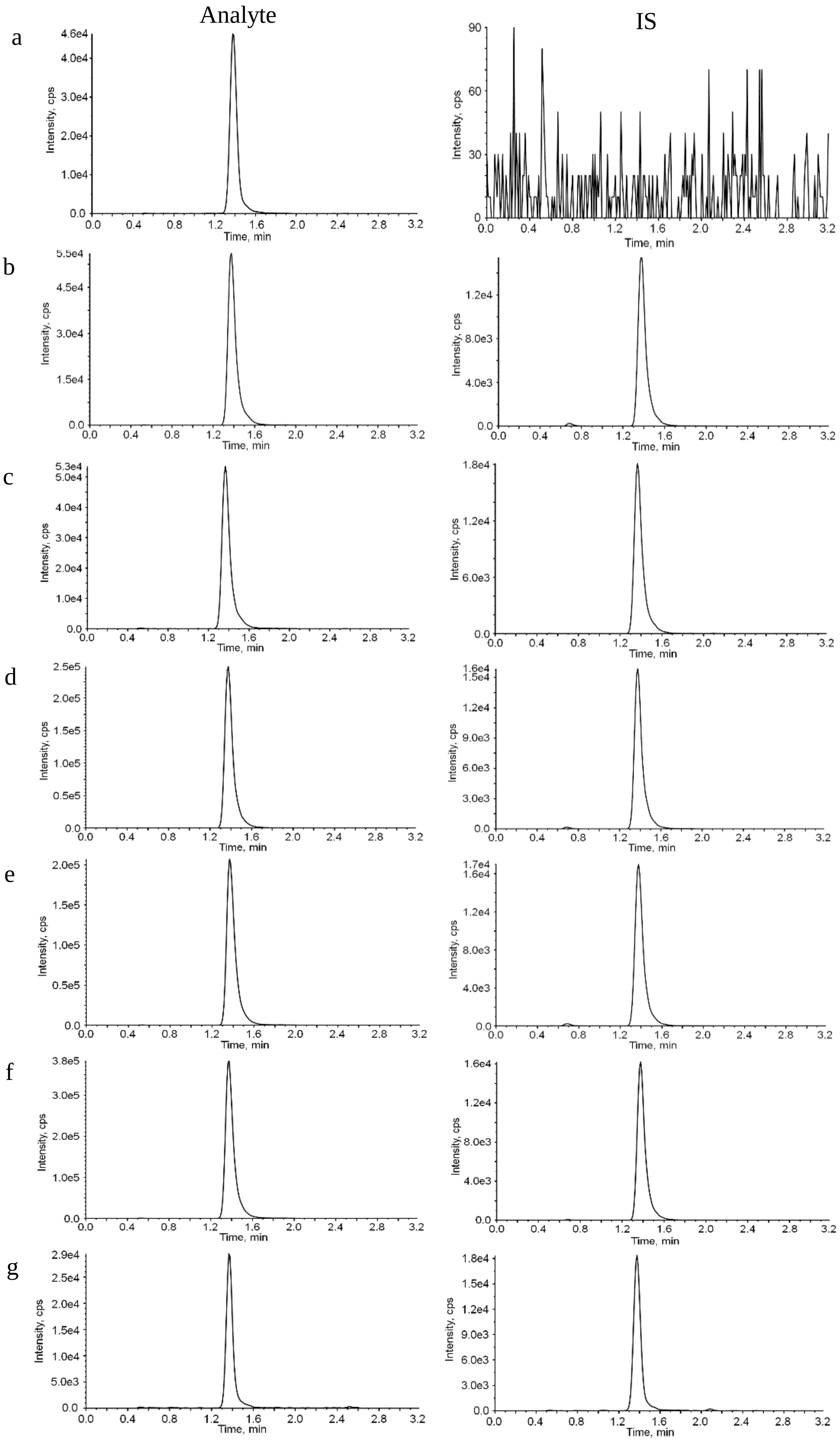

(a) blank plasma. (b) blank plasma sample spiked with sodium sulfide (1.25 µM) and ^{36}S-labeled sodium sulfide (0.313 µM). (c) plasma. (d) heart. (e) liver. (f) kidney. (g) NRCMs.

Supplementary Figure S4: Western blots of primary neonatal rat cardiac ventricular myocytes (NRCMs) after transfection of CSE siRNA.

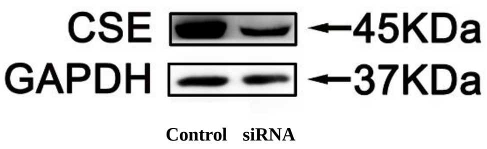

Supplementary Figure S5: Western blots of several tissues for CSE wild-type mice (*CSE*$^{+/+}$) and knockout mice (*CSE*$^{-/-}$).

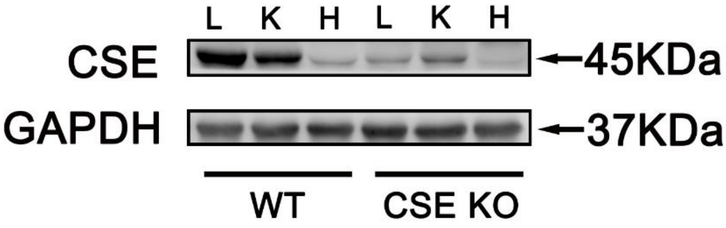

L: liver. K: kidney. H: heart.

Supplementary Table S1:

Accuracy and precision for the analysis of hydrogen sulfide (^{36}S-labeled hydrogen sulfide) in mice plasma (*n* = 3 days, six replicates per day).

	Nominal concentration (μM)	Intra-batch			Inter-batch		
		Determined concentration (mean ±SD, μM)	RE (%)	RSD (%)	Determined concentration (mean ±SD, μM)	RE (%)	RSD (%)
Plasma	0.039	0.043 ± 0.002	9.1	4.2	0.041 ± 0.002	5.8	5.5
	0.078	0.084 ± 0.008	7.5	9.0	0.080 ± 0.006	3.0	7.5
	2.5	2.47 ± 0.12	-1.1	4.8	2.49 ± 0.13	-0.6	5.2
	18	17.2 ± 0.6	-4.4	3.3	17.2 ± 0.5	-4.4	3.2
Heart	0.039	0.042 ± 0.002	7.0	5.6	0.041 ± 0.003	4.7	6.7
	0.078	0.074 ± 0.002	-5.4	2.9	0.073 ± 0.004	-6.1	4.8
	2.5	2.49 ± 0.08	-0.6	3.0	2.54 ± 0.16	1.7	6.1
	18	17.4 ± 0.6	-3.5	3.5	17.4 ± 0.6	-3.3	3.2
Liver	0.039	0.041 ± 0.003	4.4	7.8	0.040 ± 0.003	3.1	6.8
	0.078	0.073 ± 0.003	-5.9	4.4	0.074 ± 0.003	-4.7	4.4
	2.5	2.49 ± 0.10	-0.5	3.9	2.53 ± 0.15	1.3	5.9
	18	18.1 ± 1.0	0.3	5.8	18.3 ± 0.9	1.5	4.8
Kidney	0.039	0.038 ± 0.001	-3.6	3.8	0.037 ± 0.002	-4.2	5.2
	0.078	0.071 ± 0.005	-9.0	7.3	0.072 ± 0.004	-7.4	6.1
	2.5	2.32 ± 0.08	-7.2	3.4	2.34 ± 0.10	-6.6	4.3
	18	16.7 ± 0.6	-7.3	3.5	16.9 ± 0.6	-5.8	3.4
Cultured cells	0.039	0.039 ± 0.003	0.5	6.7	0.040 ± 0.002	1.6	6.2
	0.078	0.074 ± 0.004	-4.6	4.8	0.072 ± 0.003	-7.2	4.6
	2.5	2.53 ± 0.14	1.3	5.5	2.43 ± 0.16	-2.9	6.8
	18	18.6 ± 0.6	3.1	3.0	18.3 ± 0.7	1.5	3.6

Supplementary Table S2:

Recovery and matrix effect of hydrogen sulfide (^{36}S-labeled hydrogen sulfide) in different mice tissues (*n* = 6).

	Statistical variable	Nominal concentration (μM)		
		0.078	2.5	18
Plasma	Recovery			
	Mean(%)	73.5	77.8	79.3
	RSD(%)	4.4	2.6	3.0
	Matrix effect			
	Mean(%)	86.8	91.7	89.2
	RSD(%)	7.4	5.0	5.8
Heart	Recovery			
	Mean(%)	73.5	76.5	78.1
	RSD(%)	4.8	4.5	3.6
	Matrix effect			
	Mean(%)	80.9	85.9	86.2
	RSD(%)	6.2	6.2	5.9
Liver	Recovery			
	Mean(%)	71.4	76.1	77.3
	RSD(%)	6.2	6.1	5.3
	Matrix effect			
	Mean(%)	81.2	84.9	86.3
	RSD(%)	7.1	4.4	6.9
Kidney	Recovery			
	Mean(%)	72.4	75.7	76.3
	RSD(%)	5.4	4.2	6.4
	Matrix effect			
	Mean(%)	79.6	83.9	84.8
	RSD(%)	5.7	6.2	5.8
Cultured cells	Recovery			
	Mean(%)	74.7	76.4	75.5
	RSD(%)	3.1	3.5	3.5
	Matrix effect			
	Mean(%)	90.4	91.1	92.4
	RSD(%)	4.3	4.2	5.1

Supplementary Table S3:

Stability of hydrogen sulfide derivative in rat plasma (n = 6)

	Storage conditions	Nominal concentration (ng/mL)	Determination concentration (mean ±SD, ng/mL)	RE(%)	RSD(%)
Plasma	Autosampler	0.078	0.078 ± 0.002	-0.5	2.6
	(24 h, 25 ℃)	2.5	2.63 ± 0.16	5.3	6.3
		18	17.8 ± 0.6	-1.1	3.3
	Three freeze-thaw cycles	0.078	0.081 ± 0.003	4.0	3.6
	(from –20 to 25 ℃)	2.5	2.55 ± 0.20	2.0	7.9
		18	15.8 ± 0.9	-12.0	5.1
	Long-term storage	0.078	0.075 ± 0.003	-4.1	4.4
	(13 d, –20 ℃)	2.5	2.58 ± 0.08	3.2	3.3
		18	16.3 ± 0.1	-9.3	0.7
Heart	Autosampler	0.078	0.084 ± 0.003	7.1	4.2
	(24 h, 25 ℃)	2.5	2.67 ± 0.16	6.6	6.6
		18	18.3 ± 0.3	1.8	1.8
	Three freeze-thaw cycles	0.078	0.081 ± 0.004	3.9	5.0
	(from –20 to 25 ℃)	2.5	2.53 ± 0.03	1.2	1.3
		18	17.6 ± 1.3	-2.3	7.0
	Long-term storage	0.078	0.073 ± 0.005	-6.4	5.8
	(13 d, –20 ℃)	2.5	2.53 ± 0.03	2.8	8.3
		18	16.8 ± 0.6	-6.7	3.6
Liver	Autosampler	0.078	0.076 ± 0.003	-2.0	4.0
	(24 h, 25 ℃)	2.5	2.59 ± 0.04	3.5	1.8
		18	16.8 ± 0.4	-6.7	2.3
	Three freeze-thaw cycles	0.078	0.075 ± 0.004	-3.6	4.7
	(from –20 to 25 ℃)	2.5	2.53 ± 0.03	1.2	1.3
		18	17.0 ± 2.0	-5.4	11.0
	Long-term storage	0.078	0.075 ± 0.006	-3.9	7.4
	(13 d, –20 ℃)	2.5	2.53 ± 0.03	3.0	5.5
		18	17.1 ± 0.4	-5.2	2.1
Kidney	Autosampler	0.078	0.074 ± 0.002	-5.2	2.9
	(24 h, 25 ℃)	2.5	2.51 ± 0.13	0.4	5.0
		18	17.3 ± 0.7	-4.1	4.0
	Three freeze-thaw cycles	0.078	0.077 ± 0.002	-1.8	3.0
	(from –20 to 25 ℃)	2.5	2.53 ± 0.03	1.2	1.3
		18	17.4 ± 0.4	-3.3	2.4
	Long-term storage	0.078	0.071 ± 0.003	-9.5	4.7
	(13 d, –20 ℃)	2.5	2.53 ± 0.03	-6.3	1.3
		18	16.7 ± 0.3	-7.0	1.7
Cultured cells	Autosampler	0.078	0.074 ± 0.001	-4.9	1.2
	(24 h, 25 ℃)	2.5	2.57 ± 0.09	2.9	3.4
		18	17.7 ± 0.4	-1.6	2.5
	Three freeze-thaw cycles	0.078	0.076 ± 0.002	-2.3	2.9
	(from –20 to 25 ℃)	2.5	2.53 ± 0.03	1.2	1.3
		18	16.8 ± 0.8	-6.5	4.7
	Long-term storage	0.078	0.074 ± 0.004	-5.4	5.5
	(13 d, –20 ℃)	2.5	2.53 ± 0.03	1.5	1.3
		18	17.4 ± 0.5	-3.1	2.9
